# Within the Wards

**Published:** 1888-07-21

**Authors:** 


### Within the Wards.

### FLAT FEET.

Mr. J. S. Ellis, of Gloucester, records the case of a farrier's apprentice who was under his care in the Gloucester Infirmary fifteen years ago. His feet were at that time so flat and painful that he could not walk across the ward. He was put in the way of proper exercises, and ultimately became a distinguished dancer in the 2nd Life Guards. The best exercise for the cure of flat-foot Mr. Ellis considers to be the raising of a weight by means of a cord running over pulleys. The same exercise is suitable for many cases of knock-knee. The object is to bring the foot to extreme tiptoe, and the knee and hip to full extension. This is to be done simultaneously with the act of inspiration, so as to get the benefit of the Silvester method of artificial respiration added to that of the natural breathing. When full tiptoe has been reached there should be a pause, followed by a sudden and vigorous drawing downwards. The turning of a wheel, placed so high that the handle is with difficulty reached when at the highest part of the cycle, is a similar movement, and has the advantage of being less irksome than the cord and pulley method. Pumping from a deep well, if the pump handle be placed at the proper elevation, and bell-ringing are also exercises excellently adapted to benefit those who have flat feet or knock knees. Persons who are constantly engaged in certain departments of woodman's or gardener's work, such as reaching up to and drawing down the boughs of trees, are believed to be unusually free from flat-footedness. To these exercises are to be added a plentiful dietary, and stimulation of muscular nutrition by friction, massage, and other such means.

### PARALYSIS AFTER DIPHTHERIA.

It is a familiar experience with medical men for some form of local paralysis to follow an attack of diphtheria. The heart may be affected, during or after convalescence, with alarming and sometimes fatal results. Or the organs of voice and speech may suffer, and a prolonged period of comparative disability ensue. Or the eye may be attacked in some of its structure, and impairment or loss of sight appear imminent. The severity of the throat symptoms is no constant index of the degree of paralysis which may follow, or of the muscle or muscles which may be selected for attack. Mr. Jonathan Hutchinson states that paralysis of the muscles of accommodation, such as is usually described as "diphtheritic," may occur after very slight forms of sore throat. He has seen and recorded several such cases, in which the sore throat had been so slight and transitory that it had been forgotten. He says: "A healthy-looking girl was brought to me on account of failure of sight, which I at once found to be paralysis of accommodation. I inquired as to the sore throat, and was told by her mother that the child had not been ill in any way. At the second visit, however, two facts were remembered which had been at first forgotten. One month before coming to me the child had been in company with a little boy who had been suffering from a bad sore throat, and some time ago had had diphtheria. Three weeks prior to this, however, the child herself, while staying at Sandown, had had a three days' sore throat; but it had not been thought bad enough to necessitate medical advice. Such were the (apparently) trivial, but I have no doubt, really important facts. The child under treatment recovered perfectly in about three weeks, and had no other symptoms of paralysis. I cannot doubt that the failure of sight was of the nature of diphtheritic paralysis. Does the case prove that true diphtheria may occasionally be so slight as to be overlooked, and yet be efficient to the production of paralysis as a sequela? This is my own belief, and it seems to me to establish a very important fact with reference to the natural history of the disease. I believe it is generally acknowledged that the severity of diphtheritic paralysis is not usually in the same ratio with the severity of the throat affection."

### DIARRHŒA TREATED BY SALOL.

Dr. O. J. Osborne, in the *New York Medical Record*, reports a number of cases of diarrhœa in children and adults in which salol produced most satisfactory results. To a child under two years of age he gave about $\frac{3}{4}$ gr.; from two to five years, $1\frac{3}{4}$ grs.; from five to twelve years, 3 grs.; and to all above twelve years, from 4 grs. to 5 grs. The last dose he found to be usually quite sufficient for adults. He considers it necessary for immediate success to repeat the dose every two hours. The indications for salol seem to be vomiting, purging, and cramps. The summer diarrhœas of children are said to yield readily to this treatment, and also chronic diarrhœa and dysentery have been benefited by it. All Dr. Osborne's cases, except one dysenteric case, were cured without opium in any form, and with no other drug than salol.

### THE BACILLUS OF CANCER.

The mind of man is never content until it ascertains the causes of things. Being a mind of strictly limited capacity it sometimes performs decidedly amusing antics in its search after causes. The bacillus has been a fortunate "find" for men who desire to obtain a scientific reputation at a small cost of trouble and capacity. Many such men think that the discovery of a new species of this wonderful genus would lead them to the very summit of fame's monument in a moment. No doubt the different kinds of bacilli are responsible for an unpleasant series of circumstances, both in the human organism and elsewhere. But the bacillus suffers much injustice, like many higher creatures, from a bad reputation. He is a "dog with a bad name," and everybody is anxious, first to discover, and then no doubt to hang him; but to discover him at any rate, for the "hanging" or other process of destruction is found a little difficult. Some time ago Scheurlen was under the impression that he had discovered a bacillus whose activity was the cause of the growth and development of cancer. If that were so, a cultivation made with a normal and non-ulcerated portion of cancerous tissue should of course reproduce its like. Dr. A. Pfeiffer of Weisbaden attempted to procure cultivations on material from non-ulcerous carcinomata, but failed. The nutrient material generally remained sterile, or if a growth took place at all it was one of the well-known forms of decomposition bacteria. The *proteus mirabilis* corresponded best with Scheurlen's bacillus. Professor Baumgarten of Königsberg, a distinguished bacteriologist, recently made some investigations in association with Dr. Rosenthal. The investigators experimented on carcinomatous structures taken from the mamma and elswhere. They procured a bacillus resembling the potato bacillus on the one hand, and Scheurlen's bacillus on the other. A similar bacillus was obtained from the juice of a sarcoma of the mamma, from a sarcoma of the scalp, and from a neuroma. It was therefore not exclusively a carcinoma bacillus. Not only so, but its presence was not constant in carcinomata, for in a case of cancer of the rectum, and also in one of mammary cancer, the most rigid search failed to find it. This being so it cannot be said to be in any sense proved that Scheurlen's bacillus is the exciting cause of cancer. Baumgarten and Rosenthal are inclined to the opinion that the bacillus in question is a kind of potato bacillus that grows upon the cut surface and forces its way into the structures beneath.

